# Heterogeneous biomedical entity representation learning for gene–disease association prediction

**DOI:** 10.1093/bib/bbae380

**Published:** 2024-08-18

**Authors:** Zhaohan Meng, Siwei Liu, Shangsong Liang, Bhautesh Jani, Zaiqiao Meng

**Affiliations:** School of Computing Science, University of Glasgow, 18 Lilybank Gardens, Glasgow G12 8RZ, UK; School of Natural and Computing Science, University of Aberdeen King’s College, Aberdeen, AB24 3FX, UK; Machine Learning Department, Mohamed bin Zayed University of Artificial Intelligence, Building 1B, Masdar City, Abu Dhabi 000000, UAE; School of Computing Science, University of Glasgow, 18 Lilybank Gardens, Glasgow G12 8RZ, UK; School of Computing Science, University of Glasgow, 18 Lilybank Gardens, Glasgow G12 8RZ, UK

**Keywords:** gene, disease, fusion module, pre-training, contrastive learning, pre-trained language model

## Abstract

Understanding the genetic basis of disease is a fundamental aspect of medical research, as genes are the classic units of heredity and play a crucial role in biological function. Identifying associations between genes and diseases is critical for diagnosis, prevention, prognosis, and drug development. Genes that encode proteins with similar sequences are often implicated in related diseases, as proteins causing identical or similar diseases tend to show limited variation in their sequences. Predicting gene–disease association (GDA) requires time-consuming and expensive experiments on a large number of potential candidate genes. Although methods have been proposed to predict associations between genes and diseases using traditional machine learning algorithms and graph neural networks, these approaches struggle to capture the deep semantic information within the genes and diseases and are dependent on training data. To alleviate this issue, we propose a novel GDA prediction model named FusionGDA, which utilizes a pre-training phase with a fusion module to enrich the gene and disease semantic representations encoded by pre-trained language models. Multi-modal representations are generated by the fusion module, which includes rich semantic information about two heterogeneous biomedical entities: protein sequences and disease descriptions. Subsequently, the pooling aggregation strategy is adopted to compress the dimensions of the multi-modal representation. In addition, FusionGDA employs a pre-training phase leveraging a contrastive learning loss to extract potential gene and disease features by training on a large public GDA dataset. To rigorously evaluate the effectiveness of the FusionGDA model, we conduct comprehensive experiments on five datasets and compare our proposed model with five competitive baseline models on the DisGeNet-Eval dataset. Notably, our case study further demonstrates the ability of FusionGDA to discover hidden associations effectively. The complete code and datasets of our experiments are available at https://github.com/ZhaohanM/FusionGDA.

## Introduction

In intricate human genetics, genes are the basic units of inheritance and components of biological function. Mutations in these critical protein sequences disrupt biological processes causing disease [1]. In light of the growing number of new diseases and the accelerating demand for novel therapies, it is essential to explore the associations between known diseases and genes to infer new associations between genes and diseases [2]. By investigating existing gene–disease association (GDA), researchers have leveraged known genes or diseases as a basis for exploring associations with similar genes or conditions [3, 4]. These research efforts are foundational for understanding the underlying biological mechanisms of disease pathogenesis and subsequently for identifying novel drug targets and optimizing therapeutic strategies [5]. With the accumulation of biological data and advancements in machine learning (ML) algorithms, there is a growing interest in integrating genomics and therapeutic features to establish gene–disease network models for predictive analysis on GDA.

Traditional ML involves algorithms that enable computers to learn from and make predictions based on historical data, which has garnered extensive applications in GDA prediction [6–8]. Popular methods include support vector machines [9], decision trees [10], and k-nearest neighbors algorithms [11], which have been effectively utilized in related studies. An early notable technique involved inductive matrix completion, which utilizes features from multiple data sources to predict GDA, with the capability to handle diseases not encountered during training [6]. Subsequently, traditional ML incorporates deep collaborative filtering (DCF) and positive unlabeled learning, enhancing the model’s ability to process noisy and incomplete data [7]. Furthermore, traditional ML is also used to predict associations between specific diseases and genes, prioritizing inflammatory bowel disease (IBD) genes, as well as scoring large sets of genes by training on genes known to be associated with IBD in genomic studies [8]. Although effective, traditional ML models exhibit limitations due to insufficient training data, restricting their ability to learn a broader spectrum of knowledge. Besides, these models typically rely on linear interactions and thus may not be able to fully utilize the semantic information in protein sequence and text data. In contrast, pre-trained language models (PLMs) [12, 13] offer a solution by generating semantic representations to address these challenges in gene-disease network analysis.

Furthermore, with the advancements in graph representation learning (GRL) techniques, different graph-based methods have been proposed to boost the performance of GDA prediction. GRL methods can transform biology relational graphs into vector representations that capture the intricate relationships between nodes and edges, as well as their associated attributes [14–17]. Specifically, a heterogeneous network is constructed to combine diverse information, thereby improving the accuracy of GDA prediction [18]. Moreover, there is a relational graph neural network (GNN) that excels in handling sparse and incomplete data by utilizing privileged information paradigm [4, 19]. More recently, a parallel graph transformer network has been employed to build heterogeneous networks with diseases, genes, ontologies, and phenotypes [20], which improves the prediction performance. Similarly, a heterogeneous GRL method that contains multiple types of biological entities is proposed to preserve structure network embedding for more effective GDA prediction [21]. Although effective, GRL methods often struggle with fusing different biological entities such as protein sequence and disease descriptions, particularly lacking in-depth contextual understanding and semantic interpretation. Therefore, the integration of a fusion module and PLMs is a promising solution; for instance, as demonstrated by ProtST [22], which demonstrates the effectiveness of combining a fusion module with PLMs in downstream tasks of protein sequences and their corresponding textual information. The fusion module adeptly captures the nuanced relationships between different biological data modalities.

To address the aforementioned challenges in GDA prediction, we propose a novel model named FusionGDA, which can effectively fuse gene and disease representations, employing a pre-training phase that leverages the strengths of two PLMs as encoders: ESM-2b [12] and PubMedBERT [13]. ESM-2b, a protein language model [12, 23], excels in generating comprehensive embeddings from protein sequences by learning from extensive evolutionary data. PubMedBERT is a biomedical textual language model [13, 24] pre-trained on an extensive corpus of medical literature, which has demonstrated success in generating robust representations of medical entities. Specifically, both encoders receive pairs of protein sequences and disease descriptions, respectively, generating unimodal representations.

To overcome the limitation of GRL in integrating diverse biomedical features, our FusionGDA model incorporates a fusion module, which consists of self-attention and cross-attention mechanisms [25] to ensure that each residue and word in these patterns refine its representation by interacting with all relevant tokens. The fusion module receives the unimodal representations of genes and diseases from the protein encoder and text encoder to generate multi-modal representations. Such a module contributes to the enhancement of accuracy and robustness in the GDA prediction task. In addition, the fusion module not only mitigates the risk of overfitting by robust representations but also facilitates the fusion of heterogeneous data types without compromising model generalizability.

Moreover, we first pre-train our FusionGDA model using DisGeNET [26], a comprehensive dataset containing disease-related human genetic information. Such a pre-training phase effectively addresses the limitations of traditional ML, which struggles with insufficient training data and fails to capture deep semantic information. Besides, we use the contrastive loss as a pre-training head for this phase, where the contrastive learning loss aims to optimize the feature space so that the spatial distance between similar instances is reduced and the spatial distance between dissimilar instances is expanded [27]. Pre-training contributes to model robustness, enabling it to generalize better across diverse data types and tasks, even when the data are imbalanced. Furthermore, our pre-training phase is efficient in augmenting the model performance, which also proves its potential in addressing a diversity of tasks with similar structural modeling.

Finally, we conduct case studies on Alzheimer’s disease and stomach cancer, two high-profile diseases. For these studies, we utilize our pre-trained FusionGDA to identify genes potentially linked to these conditions. Experimental results demonstrated that our FusionGDA can successfully identify 10 genes that are most likely associated with Alzheimer’s disease and stomach cancer, respectively. The main contributions of our study include:

- We propose FusionGDA, a novel model to predict GDA by leveraging the fusion module to enrich the gene and disease representations encoded by PLMs.
- We propose to use a pre-training phase with the contrastive loss to capture the potential semantic information in a large dataset, thus generating semantically effective representations for the GDA prediction task.
- Case studies of Stomach Carcinoma and Alzheimer’s disease validate the capability of the FusionGDA model to discover disease-associated protein sequences in the context of unknown associations.

## Methodology

In this section, we first define the problem setup followed by the technical details of our proposed FusionGDA model.

### Problem definition

The challenge we are addressing is to predict associations between genes and diseases, which can be categorized as a supervised learning task. Our input consists of two distinct types of biological entities: protein sequences and disease descriptions. The objective is to accurately predict the likelihood of an association between unseen gene–disease pairs. This is formulated as estimating a probability score for each pair, expressed as $p \in [0, 1]$. These scores reflect the model’s prediction of how likely the given gene and disease have an association. The effectiveness of a model is measured by its ability to identify and quantify the degree of association between genes and disease. Therefore, it requires an architecture that can capture the complex relationships inherent in the input data and learn from them.

### Model architecture

**Figure 1:**
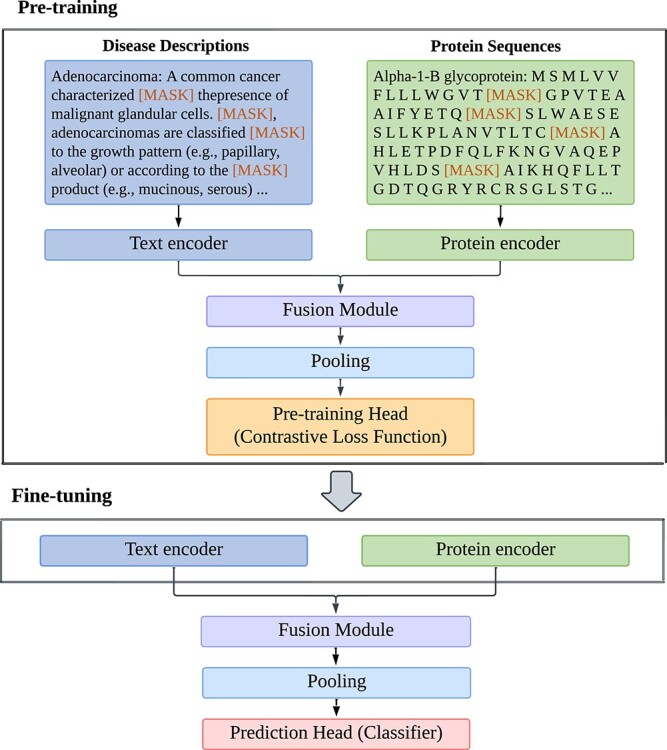
**FusionGDA**: (1) the fusion module is the cornerstone of the FusionGDA model as it allows for the synergistic combination of unimodal representations into semantically enhanced multi-modal representations of genes and diseases; this fusion approach significantly improves the ability of our proposed model to access the complex semantics underlying GDA; (2) in both the pre-training and fine-tuning phases, FusionGDA maintains a consistent structure while shifting focus from a pre-training enhancing pattern recognition of GDA to the prediction task, effectively utilizing the refined parameters for accurate predictions.

Figure 1 illustrates the overview of our proposed FusionGDA model. Specifically, FusionGDA fuses the representations of genes and diseases generated by a protein encoder (i.e. protein language model) and a text encoder (i.e. biomedical textual language model), respectively, to enhance the semantic information of the GDA predictions. Specifically, we model the gene data as a set of encoded embeddings $ G = \left [ \mathbf{G}_{1}, \mathbf{G}_{2}, \dots , \mathbf{G}_{n} \right ]$ and the disease data as $ D = \left [\mathbf{D}_{1}, \mathbf{D}_{2},...,\mathbf{D}_{m} \right ]$, where $n$ is the number of genes and $m$ is the number of diseases. Furthermore, our proposed model consists of two phases: a pre-training phase and a fine-tuning phase. In the following text, we describe the four essential components of the model including the encoders, the fusion module, the fusion representation aggregation, the pre-training head and the prediction head.

### Protein and text encoders

PLMs can be effective for biomedical downstream tasks when trained on a large corpus of relevant databases. Therefore, we utilize ESM-2b [12] as a protein encoder to encode protein sequences. Then the protein representation $\mathbf{G}$ is used as input for the fusion module. In addition, PubMedBERT [13] is a text encoder to encode disease descriptions. Disease representation $\mathbf{D}$ is also used as input to the fusion module. Overall, the rich contextual representations obtained from ESM-2b and PubMedBERT ensure that we are motivated to explore association information. Moreover, we compare some other existing protein language models (such as ProtBERT [23]) and biomedical textual language models (such as BioLinkBERT and LinkBERT [24]) in the Result and Discussion section.

### Fusion module

**Figure 2:**
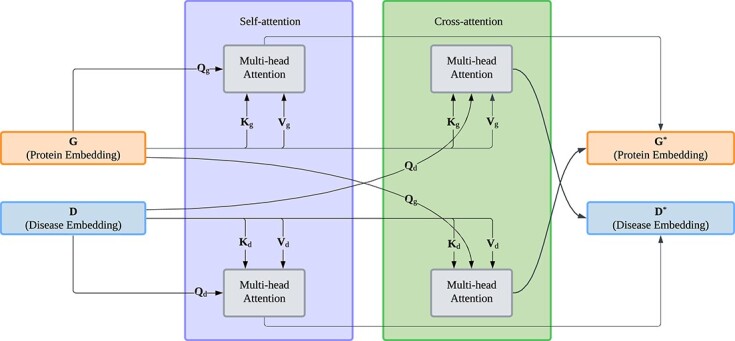
**Fusion module architecture**: the layer fuses protein representations and disease representations by querying them through self-attention and cross-attention.

In the task of GDA prediction, we focus on fusing gene and disease representations to generate fused representations that contain contextual information of both genes and diseases aiming to capture the complex interactions between them. The protein encoder and text encoder encode gene and disease entities that are inputs of our FusionGDA model to generate unimodal representations. The fusion module [22] is employed to receive uni-modal representations $ G $ and $ D $ from language models as shown in Figure 2. Its main function is to align unimodal representations of protein sequences and disease descriptions into multi-modal embeddings. In this context, the multi-head, self-attention and cross-attention mechanisms [25] are utilized to refine the representations of each residue and word as follows: 


(1) \begin{align*} \mathbf{G}^{*} &= \frac{1}{2} \left[ \text{MHA}(\mathbf{Q}_{s}, \mathbf{K}_{s}, \mathbf{V}_{s}) + \text{MHA}(\mathbf{Q}_{s}, \mathbf{K}_{t}, \mathbf{V}_{t}) \right], \end{align*}

(2) \begin{align*} \mathbf{D}^{*} &= \frac{1}{2} \left[ \text{MHA}(\mathbf{Q}_{t}, \mathbf{K}_{t}, \mathbf{V}_{t}) + \text{MHA}(\mathbf{Q}_{t}, \mathbf{K}_{s}, \mathbf{V}_{s}) \right], \end{align*}

where $ \mathbf{G}^{*} $ and $ \mathbf{D}^{*} $ represent the updated embeddings for residues and words, respectively. $\text{MHA}$ denotes the multi-head attention operation. Specifically, each fusion layer receives a sequence of residue representations and a sequence of word representations.

Figure 2 illustrates this fusion module, highlighting $ \mathbf{Q}_{s}, \mathbf{K}_{s}, \mathbf{V}_{s} $ for protein sequences and $ \mathbf{Q}_{t}, \mathbf{K}_{t}, \mathbf{V}_{t} $ for disease descriptions. These representations are then updated through both self-attention and cross-attention mechanisms to capture the interdependencies among residues and words. To facilitate this process, two distinct sets of projection matrices guide the attention mechanism [28]. Following the standard attention mechanism, we define the queries ($ \mathbf{Q}_{s} $), keys ($ \mathbf{K}_{s} $), and values ($ \mathbf{V}_{s} $) matrices of proteins as follows: 


(3) \begin{align*}& \mathbf{Q}_{s} = \mathbf{G}_{s} \mathbf{W}_{q}^{s}, \quad \mathbf{K}_{s} = \mathbf{G}_{s} \mathbf{W}_{k}^{s}, \quad \mathbf{V}_{s} = \mathbf{G}_{s} \mathbf{W}_{v}^{s}.\end{align*}

For disease representation, the matrices are constructed similarly: 


(4) \begin{align*}& \mathbf{Q}_{t} = \mathbf{D}_{t} \mathbf{W}_{q}^{t}, \quad \mathbf{K}_{t} = \mathbf{D}_{t} \mathbf{W}_{k}^{t}, \quad \mathbf{V}_{t} = \mathbf{D}_{t} \mathbf{W}_{v}^{t}.\end{align*}

Notably, our implementation uses eight attention heads per fusion layer, with a single layer to constrain capacity and enhance representational power. Finally, the fused representations are processed for dimension reduction, which is then fed into the pre-training head or the prediction head.

### Fusion representations aggregation

Following the fusion module, we obtain the fused representations for protein sequences and disease descriptions, denoted as $\mathbf{G}^{*}$ and $\mathbf{D}^{*}$, respectively. These representations exist in $\mathbb{R}^{B \times T \times H}$, where $B$, $T$, and $H$ denote the batch size, token length as employed by the PLMs i.e. protein encoder and text encoder, and the size of the hidden layer, respectively. Subsequently, these multi-modal representations are refined using one of two strategies: the Classification Token (CLS) strategy [29] or the Mean Pooling (Pooling) strategy [30]. In the case of the CLS strategy, the first token of each sequence is extracted and serves as the aggregate representation. The operation is formulated as follows: 


(5) \begin{align*}& \mathbf{G}^{*}_{cls} = \mathbf{G}^{*}[:, 0], \quad \mathbf{D}^{*}_{cls} = \mathbf{D}^{*}[:, 0].\end{align*}

These compacted vectors, $ \mathbf{G}^{*}_{cls} $ and $ \mathbf{D}^{*}_{cls} $, serve as the input for the contrastive loss function. Alternatively, the Pooling strategy computes the mean of all tokens along the sequence dimension, effectively converting the sequence information into a single representative vector. The expression of the Pooling strategy is 


(6) \begin{align*}& \mathbf{G}^{*}_{pooling} = \text{mean}(\mathbf{G}^{*}, 1), \quad \mathbf{D}^{*}_{pooling} = \text{mean}(\mathbf{D}^{*}, 1).\end{align*}

Similar to the CLS strategy, these mean-pooling vectors, $ \mathbf{G}^{*}_{pooling} $ and $ \mathbf{D}^{*}_{pooling} $, are then fed into the pre-training head or the prediction head. The Pooling strategy captures a comprehensive view of sequence features by integrating information from all tokens. In contrast, the CLS strategy focuses solely on the first token for its representation. The selection between CLS and Pooling methods depends on the specific analytical requirements and the intrinsic attributes of the data being analyzed. Ultimately, both strategies aim to optimize the model’s ability to establish robust representations of genes and diseases, though they approach the goal through differing computational techniques.

### Pre-training head

**Figure 3:**
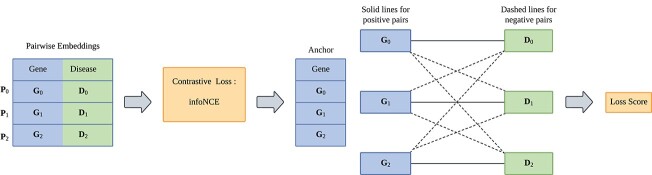
**Pre-training head**: each anchor forms a positive pair with a matching label and a negative pair with a differing label, where $\mathbf{P}_{0}, \mathbf{P}_{1}$, and $\mathbf{P}_{2}$ denote positive pairs; $\mathbf{G}_{0}, \mathbf{D}_{0}, \mathbf{G}_{1}, \mathbf{D}_{1}, \mathbf{G}_{2}$, and $\mathbf{D}_{2}$ denote the gene and disease samples; the solid lines with arrows pointing toward the anchor indicate a “pull close” action in latent space; the dashed lines with arrows pointing away from the anchor indicate a “push away” action in latent space.

The pre-training head of our model is designed to enhance the ability to identify GDAs while capturing the geometric conformation information of each residue and word. As depicted in Figure 3, the framework details how each gene embedding (anchor) forms a positive pair with its associated disease embedding and negative pairs with all other irrelevant disease embeddings. The pre-training process is conducted using a contrastive learning objective [31] designed to maximize intra-class similarities while minimizing inter-class similarities.

Through extensive testing with six different contrastive loss functions detailed in Table 3, the model adopts infoNCE as the most effective contrastive loss function [32]. InfoNCE is particularly suitable for gene and disease embeddings because it efficiently maximizes the similarity between positive pairs and minimizes that for negative pairs within a batch of samples. The mathematical formulation of the infoNCE loss is 


(7) \begin{align*}& \mathcal{L}_{\text{FusionGDA-info}} = -\log \frac{\exp \left(\mathbf{G} \cdot \mathbf{K}_{+} / \tau\right)}{\sum_{i=0}^{k} \exp \left(\mathbf{G} \cdot \mathbf{K}_{i} / \tau\right)},\end{align*}

where $ \tau $ is a temperature scaling parameter, which adjusts the probability distribution of the similarities. Here, $ \mathbf{G} $ acts as the anchor embedding, which in the case is gene-specific, and $ \mathbf K_{+} $ and $ \mathbf K_{i} $ are the embeddings of the positive and negative samples, respectively. These positive and negative samples are determined by the labels provided in the training data where each gene–disease pair with a known association is considered a positive example, and those without a known association are considered as negative examples.

### Prediction head

Building on the pre-training phase, our FusionGDA model leverages a fine-tuning phase focusing on GDA prediction with a prediction head. In this phase, LightGBM [33] is used as the classifier of choice among four candidates, as shown in Table 2. This model is selected for its proficiency in managing large datasets and categorical data, as well as for its speed and efficiency, crucial for complex tasks like GDA prediction. Specifically, LightGBM is an ensemble method that uses gradient boosting on decision trees and excels in managing high-dimensional spaces and efficiently scaling large datasets. More explicitly, the ensemble method consists of $ K $ decision trees where each tree is denoted as $ f_{k} $. The input to the classifier is composed of the pooling multi-modal representations of both genes and diseases, denoted as $\mathbf{P}^{*}_{\text{pooling}} =( \mathbf{G}^{*}_{\text{pooling}}, \mathbf{D}^{*}_{\text{pooling}} )$, which are the products of pooling. The GDA prediction score $ p \in [0, 1] $ is computed as follows: 


(8) \begin{align*}& p = \sum_{k=1}^{K} f_{k}\left(\mathbf{P}^{*}_{\text{pooling}} \right),\end{align*}

where each $ f_{k} $ maps the concatenated representations to a leaf node, the value of which contributes to the final prediction score $ p $. These scores reflect the inference of the model about potential associations between specific genes and diseases, serving as a measure of the degree of association between genes and diseases.

## Experimental setups

We use Pytorch (https://pytorch.org/) to implement our FusionGDA model and all other baselines. In addition, we use two PLMs: ESM-2b (https://huggingface.co/facebook/esm2_t33_650M_UR50D) and PubMedBERT-Fulltext (https://huggingface.co/microsoft/BiomedNLP-BiomedBERT-base-uncased-abstract-fulltext) as encoders for protein sequence and disease description loading from Hugging Face. In the pre-training phase, we will save encoders after pre-training them with a contrastive loss and the fusion module. The learning rate is set within the range of 0.0001 to 0.001 and the $ \tau $ of infoNCE is 0.07. In the fine-tuning phase, we adapt LightGBM [33] as a classifier, with the learning rate empirically set within the range of 0.1 to 0.5. We further refine the classifier by setting the maximum tree depth $ K $ to 6. This setting directly influences the number of leaves, calculated as $ 2^{K - 1} $, thereby controlling LightGBM’s complexity and performance. Furthermore, we explore different boosting strategies to optimize the classifier, including the Gradient Boosting Decision Tree (GBDT) and Random Forest [34] and Dropouts meet Multiple Additive Regression Trees (DART) [35].

### Research questions

- **RQ1**: How does the fusion module affect the performance of our proposed model?
- **RQ2**: How does the pre-training phase affect the performance of our proposed model?
- **RQ3**: Does our proposed FusionGDA model outperform the existing state-of-the-art models?
- **RQ4**: How does the FusionGDA model perform on different disease species?
- **RQ5**: Can the FusionGDA model effectively predict proteins potentially associated with Stomach Carcinoma and Alzheimer’s Disease?

### Datasets

**Figure 4:**
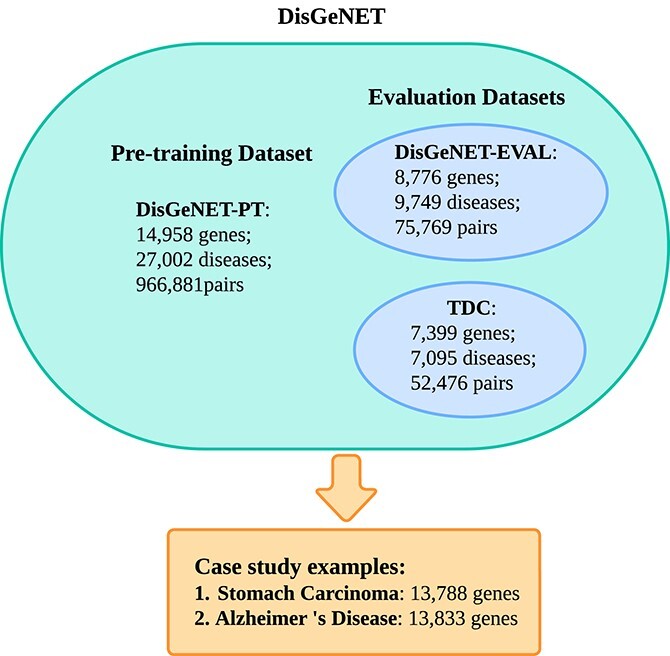
**Datasets**: **DisGeNET** is the original database of GDA from https://www.disgenet.org/; **DisGeNET-PT**, **DisGeNET-EVAL** and **TDC** are subsets of **DisGeNET**, which are used for pre-training and evaluation, respectively; **Stomach Carcinoma** and **Alzheimer’s Disease** are case study examples that obtain all pairs and genes from **DisGeNET**.

- **DisGeNET [26]** is a dataset containing information of genes and diseases, such as gene symbols, disease names, and scores indicating the association between a gene and a disease. In addition, we obtain protein sequences and disease descriptions by gene symbols and disease names of DisGeNET. In particular, disease descriptions are extracted through disease names from the Medical Genome Database Extended Form (MGDEF) [36]. For each gene, we obtain the corresponding protein sequence by matching its unique gene symbol to the STRING database [37]. In our experiment, we employ the DisGeNET (version 2023), which comprises 1041 587 GDA pairs. The dataset encapsulates 28 873 unique diseases and 16 622 distinct proteins. Specifically, our proposed model utilizes DisGeNET-PT (DisGeNET version 2022) and the DisGeNET-EVAL subset, incorporating the latest updates between versions 2022 and 2023.
- **Therapeutics Data Commons (TDC) [38]** is an open-source platform designed for computer professionals to handle multi-instance prediction tasks. TDC contains a dataset of GDAs with a 1:1 ratio of positive to negative samples. Note that all positive samples are included in the DisGeNET dataset and negative samples are randomly generated based on the positive samples.
- **Stomach Carcinoma** and **Alzheimer’s Disease** are two test datasets for case studies of specific diseases. All positive samples are obtained from the DisGeNET dataset and do not overlap with DisGeNET-PT, DisGeNET-EVAL and TDC. For negative samples, we also derive genes from DisGeNET that are not associated with either disease. Figure 4 shows the relation of these different subsets. In particular, we provide the code for all dataset generation in our GitHub.

### Baseline methods

We perform a fair comparison of baseline models using five-fold cross-validation (5-CV) [39] on the DisGeNET-EVAL in Figure 4. The results are shown in Table 4 and the following models serve as our baselines:

- **DCF [7]**: This model integrates deep representation learning and matrix completion to predict GDA.
- **HerGePred [40]:** This method utilizes random forest as its classification model. It derives node features from various heterogeneous networks and then merges the feature vectors of diseases and genes to depict the pairs of genes and diseases.
- **MHAGP [41]:** This model constructs a heterogeneous biological information network from various biomedical knowledge databases and employs GRL algorithms to extract features of gene–disease pairs. It integrates these features using multi-head attention and utilizes a multi-layer perceptron model to determine GDA.
- **GCN-MF [42]:** This framework innovatively merges the graph convolutional network (GCN) with matrix factorization techniques, enabling the effective capture of nonlinear interactions and the utilization of measured similarities. Additionally, it integrates a specially designed margin control loss function to effectively address and mitigate the challenges posed by data sparsity.
- **ERCI [43]**: This framework combines knowledge graph embedding with self-supervised learning, allowing for the generation of biological entity embeddings enriched with class information.
- **DGP-PGTN [20]**: This model is an end-to-end GDA prediction model using a parallel graph transformer network that integrates heterogeneous information on diseases, genes, ontologies, and phenotypes.

**Figure 5:**
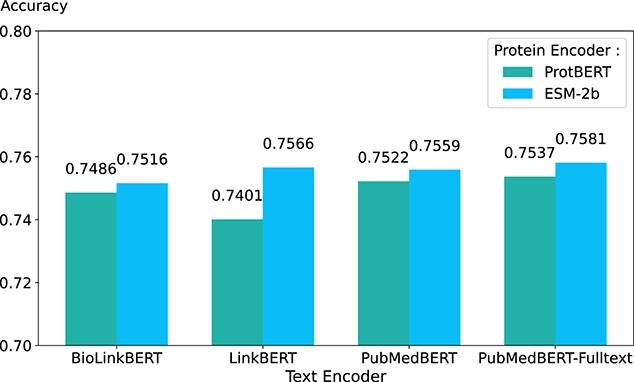
Performance comparison of two protein encoders with four different text encoders on the DisGeNET-EVAL dataset.

In addition, we explore two protein encoders and four text encoders and then compare the performance of eight different combinations as illustrated in Figure 5. The following models are included in our analysis:

- **BioLinkBERT and LinkBERT [24]**: BioLinkBERT is tailored for the biomedical domain, and fine-tuned for biomedical entity recognition and association tasks. LinkBERT is a general PLM designed to excel in understanding and generating links between different segments of text.
- **PubMedBERT and PubMedBERT-Fulltext [13]**: Variants of the BERT model have been trained on an extensive corpus of biomedical literature to capture the nuances of biomedical texts.
- **ProtBERT [23] and ESM-2b [12]**: These models are specialized in protein sequence representation and have shown promise in biomedical applications.

## Result and discussion

### Evaluation metrics

In the experimental setup, we deploy a 5-CV scheme on the DisGeNET-EVAL to assess the predictive performance of the FusionGDA model. Initially, the sample set is constructed by randomly selecting gene and disease pairs, with a particular emphasis on generating an equivalent number of negative samples that do not overlap with known GDA pairs. Following this, the amassed positive and negative samples are partitioned into five equitably sized subsets. Each subset subsequently functions as a test set, while the remaining four subsets are allocated for training.

To rigorously evaluate the predictive performance of the FusionGDA model in the context of GDA, we employ three evaluation metrics, namely ROC-AUC, AUPR, and $ \text{F}_{\text{max}} $, which are widely used to evaluate the GDA prediction performance [7, 8]. These metrics provide nuanced insights into the model’s efficacy across various aspects such as varying decision thresholds and the ranking of positive cases commonly found in GDA studies. The selection of these metrics is aligned with standard practices in the field, ensuring a comprehensive and reliable evaluation of GDA predictive models [4, 18].

1. **Receiver operating characteristic area under the curve (ROC-AUC)**: This metric assesses the accuracy of a binary classification model by comparing the true positive rate (TPR) to the false positive rate (FPR). The FPR and FPR metrics are defined as follows:
  (9) \begin{align*} \text{TPR} &= \frac{\text{True Positive}}{\text{True Positive} + \text{False Negative}}, \end{align*}
   
  (10) \begin{align*} \text{FPR} &= \frac{\text{False Positive}}{\text{False Positive} + \text{True Negative}}. \end{align*}
  ROC-AUC is formally defined as follows:
  (11) \begin{align*}& \text{ROC-AUC} = \int_{0}^{1} \mathrm{TPR}(\mathrm{FPR^{-1}(x)}) dx,\end{align*}
  where $ x $ represents the FPR at a specific point on the ROC curve. The integral calculates the area under the curve, providing a single number to summarize the model’s performance across all possible thresholds.
2. **Area Under the Precision–Recall Curve (AUPR)**: This metric is particularly useful given the class imbalance commonly observed in GDA studies. AUPR calculates the micro-average precision score for multiple binary classifications of GDA. Precision and Recall are used in its calculation and are defined as follows:
  (12) \begin{align*} \text{Precision} &= \frac{\text{True Positive}}{\text{True Positive} + \text{False Positive}}, \end{align*}
   
  (13) \begin{align*} \text{Recall} &= \frac{\text{True Positive}}{\text{True Positive} + \text{False Negative}}. \end{align*}
  The mathematical formulation for AUPR is
  (14) \begin{align*}& \text{AUPR} = \int_{0}^{1} \text{Precision}(\mathrm{Recall^{-1}(x)}) \, dx,\end{align*}
  where $ x $ represents a specific value of Recall on the Precision–Recall curve. The integral calculates the area under this curve, summarizing the model’s performance across all possible thresholds for classifying a positive sample.
3. **
$ \text{F}_{\text{max}} $ **: This metric provides a single value that balances both Precision and Recall, making it essential for a well-rounded evaluation in GDA contexts. $ \text{F}_{\text{max}} $ is defined as the maximum F-score across all possible thresholds and is calculated as
  (15) \begin{align*}& \text{F}_{\text{max}} = \max_{t \in [0, 1]} \left[ 2 \times \frac{\text{Precision}(t) \times \text{Recall}(t)}{\text{Precision}(t) + \text{Recall}(t)} \right],\end{align*}
  where $ t $ represents the decision threshold for classifying a sample as either positive or negative. The goal is to find the value of $ t $ that maximizes the F-score, effectively balancing both Precision and Recall for that particular setting.

### The effectiveness of our fusion module

For protein representation and disease representation, one simple approach would be to concatenate them together and then use a prediction head to predict GDA. However, gene and disease pairs might lack mutual contextual information about each other. Hence, we proposed a fusion module that addresses this issue using a multi-attention mechanism to enhance representations of genes and diseases. As seen in Table 1, our proposed model achieves a 3.58% improvement in AUC and a 3.25% improvement in accuracy compared with the FusionGDA-base without the fusion module. FusionGDA-base is the foundational version of FusionGDA, as shown in Figure 5 illustrating its initial framework and capabilities. Recall that the CLS strategy uses the [CLS] special token embedding to represent the entire sequence information, while the pooling strategy averages all token embeddings to form a single vector representing the sequence. As indicated in Table 1, our proposed model utilizes the fusion module with a pooling strategy, achieving a 6.9% improvement in accuracy. Therefore, in response to RQ1, the integration of the fusion module with the pooling aggregation strategy is indeed beneficial for enhancing the model’s ability to predict GDA, as evidenced by the significant improvements in AUC and accuracy over the FusionGDA-base model.

**Table 1:** Ablation study of the fusion module using an aggregation strategy (CLS or Pooling) on the DisGeNET-EVAL dataset

Fusion	Aggregation	AUC	Accuracy
$\times $	CLS	0.8734	0.7581
$\times $	Pooling	0.8877	0.7814
$\checkmark $	CLS	0.9092	0.7906
** $\checkmark $ **	**Pooling**	**0.9183**	**0.8271**

**Table 2:** Performance comparison of various prediction heads on the DisGeNET-EVAL dataset

Classifier	AUC	Accuracy
Random Forest [34]	0.9251	0.8621
eXtremGradientBoosting [44]	0.9325	0.8512
Multi-Layer Perceptron [45]	0.9321	0.8589
**LightGBM [33**]	**0.9371**	**0.8784**

### Comparison of different contrastive loss functions

By leveraging an extensive corpus of gene–disease annotations, the pre-training phase enables our proposed model to capture critical features and discern potential patterns of GDAs. As shown in Table 2, when the pre-training is paired with the LightGBM model, our FusionGDA achieves the highest performance (i.e. 0.9371 in AUC and 0.8784 in accuracy), which validates the effectiveness of pre-training phase in our FusionGDA. Moreover, the pre-training phase’s efficacy is further accentuated by the outstanding performance of the infoNCE loss function, which is chosen out of many available contrastive loss functions. As shown in Table 3, the infoNCE loss function obtains the best performance, with an accuracy of 0.8384, over all the candidate contrastive loss functions, such as MultiSimilarity [46], TripletMargin [47], and Circle [50]. This is achieved by enhancing the feature space to better distinguish between high-dimensional data points, which is crucial for the downstream task of GDA prediction. To address RQ2, we investigate the effectiveness of the pre-training phase in the FusionGDA model. Our findings indicate that the integration of the pre-training phase with the infoNCE loss function yields a synergistic effect that improves its predictive performance.

**Table 3:** Performance comparison of pre-training phase with various contrastive loss functions on the DisGeNET-EVAL dataset

Contrastive Loss	AUC	Accuracy
MultiSimilarity [46]	0.9248	0.8745
TripletMargin [47]	0.9131	0.8642
LiftedStructure [48]	0.8998	0.8247
NCA [49]	0.8954	0.8358
Circle [50]	0.8947	0.8459
**infoNCE [32]**	**0.9371**	**0.8784**

**Table 4:** Experiment results of baseline models and our FusionGDA model are compared using 5-CV on the DisGeNET-EVAL dataset

Model	AUC	AUPR	$ \text{F}_{\text{max}} $	Accuracy
DCF	0.8356	0.8195	0.8421	0.7962
HerGePred	0.8023	0.8146	0.7991	0.7846
MHAGP	0.8481	0.8515	0.8321	0.8621
GCN-MF	0.8855	0.9073	0.8794	0.8503
DGP-PGTN	0.8907	0.9045	0.8543	0.8602
ERCI	0.9055	0.8962	0.8509	0.8699
**FusionGDA**	**0.9371**	**0.9389**	**0.8727**	**0.8784**

### Comparison of different protein encoders and text encoders

The choice of protein encoder [12, 23] and text encoder [13, 24] is fundamental for pre-training and fusion of representations, as these encoders are responsible for generating representations. Our proposed model employs two PLMs encoding two biomedical entities: proteins and diseases, respectively. As seen in Figure 5, it illustrates the comparative performance of two protein encoders, ProtBERT and ESM-2b, when combined with four different text encoders: BioLinkBERT, LinkBERT, PubMedBERT, and PubMedBERT-Fulltext. Notably, the top-performing model (FusionGDA-base) combines ESM-2b and PubMedBERT-Fulltext. Therefore, the objective of this comparative analysis is to discern the most effective combination of PLMs to employ as the basis encoders for our proposed FusionGDA. Moreover, in addressing RQ3, our benchmark results in Table 4 reveal that our proposed model outperforms existing state-of-the-art models (i.e. 0.9371 in AUC and 0.9389 in AUPR), demonstrating its superior efficacy in GDA prediction.

### Performance of diseases species through ICD-10 classification

**Figure 6:**
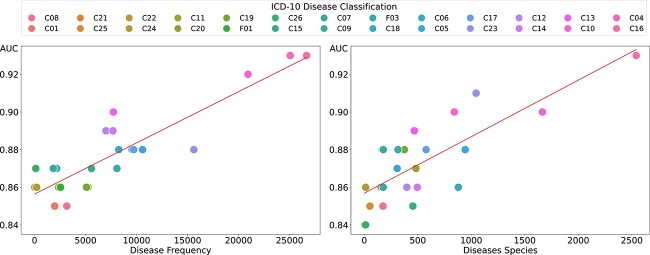
The left subplot delineates the correlation between the disease frequency and AUC values according to the ICD-10 disease classifications in the TDC dataset; the right subplot depicts the relationship between disease species and their corresponding AUC.

To investigate the predictive efficacy of the FusionGDA model, this model identifies genes associated with disease categories based on the ICD-10 classification [51]. Specifically, the pairs of genes and diseases in the TDC dataset 4 are classified into 26 subcategories, and then we examine the performance of our proposed model in different disease categories. Figure 6 highlights how the performance of our proposed model varies with disease frequency and disease species. As shown in the left figure, we observe that there is a significant positive correlation (Pearson correlation coefficient = 0.89) between the frequency of disease and the AUC values. This correlation indicates that FusionGDA trained on subcategories with a larger dataset tends to have higher predictive performance.

In the right subplot, a substantial positive correlation (Pearson correlation coefficient = 0.79) is also evident between disease species and the AUC. This observation implies that our proposed model benefits from a broader range of disease species within its training dataset, significantly enhancing its predictive power. In exploring RQ4, these findings suggest that higher disease frequency and a more diverse range of disease species in subcategories contribute significantly to the efficiency of FusionGDA in predicting GDA, as indicated by strong positive correlations in both aspects.

## Case study

### Prediction of protein sequences for stomach carcinoma and Alzheimer’s disease

To examine the predictive efficacy of our proposed model for specific disease-related proteins, we deploy the FusionGDA model to obtain high-confidence GDA pairs of Stomach Carcinoma and Alzheimer’s Disease. Specifically, we rank the prediction scores $ p $ of FusionGDA to obtain the top 10 pairs of two diseases. Focusing initially on Stomach Carcinoma, as detailed in Table 5, the top 10 protein sequences associated with this cancer are identified. Remarkably, eight of the top-ranked protein sequences are supported by scientific literature, highlighting their relevance, such as “cadherin 1” and “fibroblast growth factor receptor 2.” However, two predictions, specifically “tumor necrosis factor” and “epidermal growth factor receptor,” lack published evidence, presenting them as prospective subjects for further scientific enquiry.

**Table 5:** Top 10 protein sequences associated with Stomach Carcinoma; the term “PMID” denotes PubMed Identifier, a unique number for scientific papers in PubMed that confirms the GDA; “Unknown” indicates that the association has not yet been substantiated by evidence

Rank	Gene name	Evidence
1	cadherin 1	PMID:31077828 (2020)
2	interferon regulatory factor 1	PMID:31186404 (2019)
3	fibroblast growth factor receptor 2	PMID:30662521; 31258762; 31396354 (2019)
4	erb-b2 receptor tyrosine kinase 2	PMID:31633500; 31667572 (2020)
5	mutY DNA glycosylase	PMID:25820570 (2015)
6	caspase 10	PMID:11973654; 11973654 (2002)
7	MET proto-oncogene, receptor tyrosine kinase	PMID:30455128; 31799656 (2019)
8	tumor necrosis factor	Unknown
9	erb-b2 receptor tyrosine kinase 3	PMID:30621788; 31484705 (2019)
10	epidermal growth factor receptor	Unknown

Turning to Alzheimer’s Disease, Table 6 enumerates the top 10 protein sequences predicted by FusionGDA. Among them, seven genes, including “angiotensin I converting enzyme” and “amyloid beta precursor protein,” have been corroborated by scholarly articles. In addition, the genes that are predicted to be associated with Alzheimer’s disease such as “synthesis of cytochrome C oxidase 2,” “ALG9 alpha-1,2-mannosyltransferase,” and “ring finger protein 113A” remain unknown, but our proposed model suggests directions for future research. Therefore, in response to RQ5, this case study demonstrates that our FusionGDA model not only effectively predicts proteins associated with specific diseases, but also has the potential to uncover hidden associations, thereby aiding biomedical research.

**Table 6:** Top 10 protein sequences associated with Alzheimer’s disease

Rank	Gene name	Evidence
1	angiotensin I converting enzyme	PMID:30991105; 31734340 (2020)
2	amyloid beta precursor protein	PMID:31654319; 31605298 (2020)
3	triggering receptor expressed on myeloid cells 2	PMID:31217084; 31727362 (2020)
4	apolipoprotein E	PMID:31815697 (2020)
5	microtubule associated protein tau	PMID:31715291; 31813628 (2020)
6	insulin receptor	PMID:31823898 (2020)
7	synthesis of cytochrome C oxidase 2	Unknown
8	ALG9 alpha-1,2-mannosyltransferase	Unknown
9	ring finger protein 113A	Unknown
10	brain derived neurotrophic factor	PMID:31625272; 31518516 (2020)

## Conclusion

With the rapid discovery of new diseases and the urgent need for innovative therapies, it is critical to understand the complexity of GDA. The current strategies, which employ both GNN approaches and traditional ML techniques, focus on analyzing the associations between known genes and diseases. This knowledge is then utilized to infer potential associations with newly identified diseases or genetic variants. The underlying assumption of this approach is that proteins with similar sequences may be associated with the same diseases. However, these techniques often fail to capture the intricate semantic associations between genes and diseases, and their efficacy is highly dependent on the quality of the training data. To address these challenges, we introduce FusionGDA, a novel pre-trained model that innovatively fuses heterogeneous biomedical representations to enhance GDA predictions. Drawing on the strengths of both protein encoder and text encoder, the FusionGDA model synergistically combines these modalities to deepen the contextual understanding of gene and disease entities. At the heart of our model lies the fusion module, which adeptly synthesiszs multi-modal representations, thereby amplifying the informational essence of each gene and disease entity.

Furthermore, we have integrated a contrastive loss function during pre-training as a pivotal optimization mechanism. In the fine-tuning phase, our proposed model harnesses the predictive power of LightGBM, serving as a sophisticated classification head, to deliver precise and reliable GDA predictions. The efficacy of FusionGDA is underscored by its performance metrics, with an AUC value of 0.9371, an AUPR value of 0.9389, and an $ \text{F}_{\text{max}} $ value of 0.8727, as evaluated on the DisGeNET datasets. To rigorously assess the predictive capabilities of our proposed model, we construct separate test sets targeting two specific conditions: Stomach Carcinoma and Alzheimer’s disease. These targeted evaluations demonstrate the model’s proficiency in identifying relevant associations. Further supporting its potential, case studies indicate that FusionGDA serves a crucial role in identifying new therapeutic targets, showcasing its applicability in the sphere of novel discovery.

**Key Points**

- We propose FusionGDA, a novel model for predicting GDA that uses a fusion module to enrich the semantic information of genes and diseases encoded in PLMs.
- In the pre-training phase, FusionGDA employs a contrastive loss function to build foundational knowledge and robust feature representations of gene–disease pairs from a large dataset.
- In the fine-tuning phase, our proposed model utilizes an efficient prediction head to enhance the prediction accuracy, leveraging gradient boosting on decision trees.
- Through case studies on Stomach Carcinoma and Alzheimer’s Disease, we demonstrate that our proposed model is able to discover potential proteins associated with these conditions.

## Data Availability

The data used are publicly available datasets and relevant citations and descriptions are included in our paper.
